# Improving Direct Yaw-Moment Control via Neural-Network-Based Non-Singular Fast Terminal Sliding Mode Control for Electric Vehicles

**DOI:** 10.3390/s24134079

**Published:** 2024-06-23

**Authors:** Jung Eun Lee, Byeong Woo Kim

**Affiliations:** Department of Electrical, Electronic and Computer Engineering, University of Ulsan, Ulsan 44610, Republic of Korea; rotnn429@ulsan.ac.kr

**Keywords:** direct yaw-moment control, non-singular fast terminal sliding mode control, radial basis function neural network, yaw stability, four-wheel independent drive electric vehicle

## Abstract

Given the increased significance of electric vehicles in recent years, this study aimed to develop a novel form of direct yaw-moment control (DYC) to enhance the driving stability of four-wheel independent drive (4WID) electric vehicles. Specifically, this study developed an innovative non-singular fast terminal sliding mode control (NFTSMC) method that integrates NFTSM and a fast-reaching control law. Moreover, this study employed a radial basis function neural network (RBFNN) to approximate both the entire system model and uncertain components, thereby reducing the computational load associated with a complex system model and augmenting the overall control performance. Using the aforementioned factors, the optimal additional yaw moment to ensure the lateral stability of a vehicle is determined. To generate the additional yaw moment, we introduce a real-time optimal torque distribution method based on the vertical load ratio. The stability of the proposed approach is comprehensively verified using the Lyapunov theory. Lastly, the validity of the proposed DYC system is confirmed by simulation tests involving step and sinusoidal inputs conducted using Matlab/Simulink and CarSim software. Compared to conventional sliding mode control (SMC) and NFTSMC methods, the proposed approach showed improvements in yaw rate tracking accuracy for all scenarios, along with a significant reduction in the chattering phenomenon in control torques.

## 1. Introduction

With environmental issues exacerbated by climate change and atmospheric pollution persisting, countries are implementing diverse policies and regulations for environmental protection. Electric vehicles (EVs) are recognized as an environmentally friendly transportation option and a viable alternative to address environmental concerns [1]. With growing interest in EVs, the automotive industry is actively engaged in research and development to advance their related technologies. Various EV designs are currently in development to cater to diverse vehicle needs and customer preferences, which, in turn, spurs technological advancements [2,3]. Notably, the 4WID configuration, featuring an electric motor installed in each wheel, offers enhanced energy efficiency and a compact structure owing to shorter power transmission units enabled by an in-wheel motor that independently controls each wheel [4]. Moreover, the 4WID configuration allows for faster and more precise torque and speed control, garnering significant attention.

Real-world driving situations are notably complex, often involving high speeds and sudden turns, which can lead to instability if the vehicle lacks sufficient lateral force [5]. Thus, ensuring effective steering control and managing lateral force are critical concerns for enhancing driver comfort and vehicle stability during driving. Common vehicle position control mechanisms include the active front steering (AFS) [6,7], the DYC [8,9,10], and the anti-lock braking system (ABS) [11,12]. Among these, DYC not only improves the driving comfort compared to AFS and ABS but also significantly contributes to stabilizing the vehicle movement, particularly at high speed and during sudden turns. These advantages have attracted considerable research attention in recent years. The fundamental concept of DYC involves modifying a vehicle’s yaw movement by applying an extra yaw moment, which is determined according to data gathered from the steering wheel angle. This additional yaw moment is generated by the driving or braking force exerted by each wheel, a process that can be easily and conveniently executed through the 4WID configuration.

Ensuring smooth control and performance reliability poses challenges for DYC systems due to the non-linearity, uncertainties, and interconnected dynamics of vehicles. Consequently, significant research endeavors are underway to address these challenges through various control strategies, including proportional–integral–derivative (PID) control [13,14], linear quadratic regulator (LQR) control [15,16], model predictive control (MPC) [17,18], and resilient control [19,20]. Initially, studies emphasized the implementation of a PID controller [13,14]. Although PID controllers offer simplicity in design and quick response, their performance diminishes under varying driving conditions due to their design assumptions of system linearity. Additionally, achieving optimal gain tuning poses a significant challenge. Recent advancements have introduced linear control methods like the LQR [15,16] to enhance control performance. However, these techniques also presuppose system linearity and assume the measurement of all states without external interference. Consequently, these control systems remain vulnerable to disturbances from the external environment or inaccuracies in modeling real-world systems, particularly in segments demonstrating significant non-linearity. Moreover, MPC [17,18] is commonly used for longitudinal tracking in pure electric vehicles. However, recent MPC methods do not typically address the issue of softened constraints. MPC often imposes strict constraints on states, outputs, and inputs, which can result in the inability to find a feasible solution, potentially leading to instability. Additionally, MPC requires an accurate system model, which can be difficult to obtain due to the inherent uncertainties and non-linearities of vehicle dynamics. Therefore, further research should focus on enhancing the accuracy of modeling non-linearity in vehicles and tires, as well as implementing more sophisticated algorithms.

Recently, SMC has gained recognition for its effectiveness in handling uncertainties, leading to its widespread adoption in DYC system design [21,22,23]. The conventional SMC approach is robust against uncertainty, enhances system performance through rapid response, and is straightforward to implement. Due to advancements such as the recent proposal of NFTSMC, there have been enhancements in the convergence speed, accuracy, and robustness of motion tracking, along with the resolution of singularity issues [24,25,26]. Nonetheless, SMC-based controllers unavoidably exhibit chattering due to the presence of sign function in the reaching control law. The chattering phenomenon causes wear in components and can rapidly degrade the system, posing a threat to the vehicle system. Furthermore, establishing upper limits for uncertainties in vehicle dynamics is necessary, although determining an appropriate upper limit can occasionally be challenging. Likewise, SMC-based methods still face the challenge of performance being constrained by the real system conditions [23].

In recent years, to address challenges related to the computational complexity of system models and their inherent uncertainties, research has predominantly focused on techniques like fuzzy logic systems [27,28,29] and neural networks (NNs) [14,30]. A previous study introduced a fuzzy-based adaptive PID path-tracking control method [27] that adjusts PID control parameters based on lateral variation and its rate of change. Another study [14] presented an NN PID controller designed for lateral path tracking control, utilizing steering system models, achieving robustness by updating control parameters using an NN. While an NN-based feedback control undeniably enhances the stability of electric vehicles, it still falls short of meeting certain requirements in extreme scenarios. Recent studies have introduced several NN-based control mechanisms, including feed-forward NNs, recurrent NNs, and radial basis function NNs (RBFNNs), aiming to estimate unknown uncertainties or dynamics models of vehicle systems. RBFNNs, in particular, have shown promise in enhancing control performance efficiently [30,31,32,33,34]. The weight update rule of RBFNNs is derived from the Lyapunov theory, ensuring control performance through global stability and convergence. Additionally, learning processes significantly reduce the chattering phenomenon and lead to more accurate predictions of vehicle non-linearity.

Building on the prior research, this study aims to improve the lateral driving stability by introducing a novel DYC strategy designed to withstand external disturbances and uncertainties within the vehicle system. The contributions of this study are as follows:

- Unlike recent studies that develop controllers based on SMC [35], TSMC [22], or NTSMC [36] for 4WID EVs, this paper introduces an innovative NFTSMC method. This is achieved through the integration of an NFTSM surface and a fast-reaching control law. This approach not only circumvents the singularity issue in control input but also guarantees rapid reduction of the tracking error towards zero compared to methods [22,35,36].
- In contrast to [22,35,36], which require calculating the exact system model, this research employs an RBFNN to approximate the entire system model and its uncertain components. Through this design, the proposed control method offers a novel model-free solution for 4WID EVs, eliminating the need to consider the system model while computing the control signal. This makes the approach easily applicable to real systems. Furthermore, by leveraging accurate information from the RBFNN, it significantly enhances tracking performance and effectively reduces chattering behavior in control signals.
- The stability of the proposed method has been thoroughly verified using the Lyapunov theory, ensuring its reliability across various conditions.
- During the verification process carried out via test simulations using CarSim and Matlab software, a significant enhancement in yaw rate tracking accuracy was observed, along with a notable reduction in the chattering of the input control signals.

The rest of this paper is structured as follows. Section 2 presents the vehicle dynamics models used for computing reference values and designing the controller. Section 3 details the design of the proposed control method for the upper level of the DYC system and introduces a torque distribution method based on the vertical load ratio to ensure smooth torque distribution across the four wheels. Section 4 discusses the simulation results that verify the effectiveness of the suggested DYC system. In conclusion, Section 5 concludes the paper.

## 2. Vehicle Dynamics

### 2.1. Vehicle Dynamics Model

**Figure 1:**
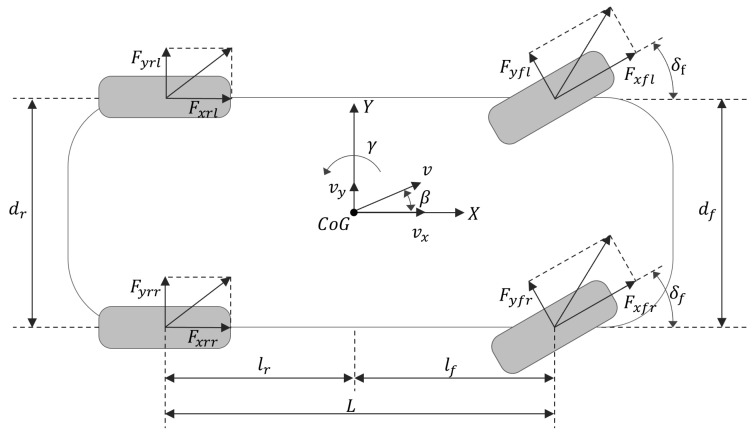
Nonlinear 7-DOF vehicle model.

Owing to the intricate mechanical design of vehicles, their dynamic systems consistently involve significant non-linearity and uncertain parameters, leading to inevitable modeling errors. In this study, a seven-degree-of-freedom (7-DOF) vehicle dynamics model, depicted in Figure 1, was developed to streamline the impact of extraneous variables and comprehensively address a range of factors.

The motion of the vehicle is characterized by longitudinal, lateral, and yaw movements, as described by the Newton–Euler equations, which account for the forces from the four tires. For design convenience, it is assumed that the front steering wheel angles on both sides are identical when the vehicle is turning. The road surface is considered flat, having no influence on the vertical movement of the wheels. Additionally, factors such as torsional vibration and shimmy vibration are not taken into account. These dynamics are detailed as follows [10]:

1. Longitudinal motion:
  (1) $$\left\{\begin{array}{c} ma_{x}=m\left({\dot{v}}_{x}-v_{x}\gamma \right)=F_{x}\\ F_{x}=F_{xrl}+F_{xrr}-\left(F_{yfl}+F_{yfr}\right)\sin\delta_{f}+\left(F_{xfl}+F_{xfr}\right)\cos\delta_{f} \end{array}\right.$$
2. Lateral motion:
  (2) $$\left\{\begin{array}{c} ma_{y}=m\left({\dot{v}}_{y}+v_{x}\gamma \right)=F_{y}\\ F_{y}=\left(F_{yfl}+F_{yfr}\right)\cos\delta_{f}-\left(F_{xfl}+F_{xfr}\right)\sin\delta_{f}+F_{yrl}+F_{yrr} \end{array}\right.$$
3. Yaw motion:
  (3) $$\left\{\begin{array}{c} I_{z}\dot{\gamma }=M_{z}\\ M_{z}=l_{f}\left[\left(F_{xfl}+F_{xfr}\right)\sin\delta_{f}+\left(F_{yfl}+F_{yfr}\right)\cos\delta_{f}\right]\;\;-l_{r}\left(F_{yrl}+F_{yrr}\right)\\ \;\;\;\;\;\;\;\;+\frac{d_{f}}{2}\left[\left(F_{xfr}-F_{xfl}\right)\cos\delta_{f}+\left(F_{yfl}-F_{yfr}\right)\sin\delta_{f}\right]+\frac{d_{f}}{2}\left(F_{xrr}-F_{xrl}\right)+D \end{array}\right.$$

Here, *m* denotes the mass of vehicle; $v_{x}$ and $v_{y}$ represent the longitudinal and lateral velocity components, respectively; $a_{x}$ and $a_{y}$ are the longitudinal and lateral acceleration components. γ, $I_{z}$, and $\delta_{f}$ denote the yaw rate, yaw inertia of each vehicle’s coordinate system, and steering angle of front wheels, respectively. $l_{f}$ and $l_{r}$ are the vertical distances from the front and rear axles to the vehicle center, respectively. $d_{f}$ and $d_{r}$ denote the treads of the front and rear wheels, respectively. Additionally, $F_{xfl}$, $F_{xfr}$, $F_{xrl}$, and $F_{xrr}$ are longitudinal force components of each tire, while $F_{yfl}$, $F_{yfr}$, $F_{yrl}$, and $F_{yrr}$ are lateral force components of each tire. $M_{z}$ is the yaw moment, *D* denotes the uncertainty due to external disturbance and the system model itself, where $\left|D\right|\leq \bar{D}$, and $\bar{D}>0$.

**Figure 2:**
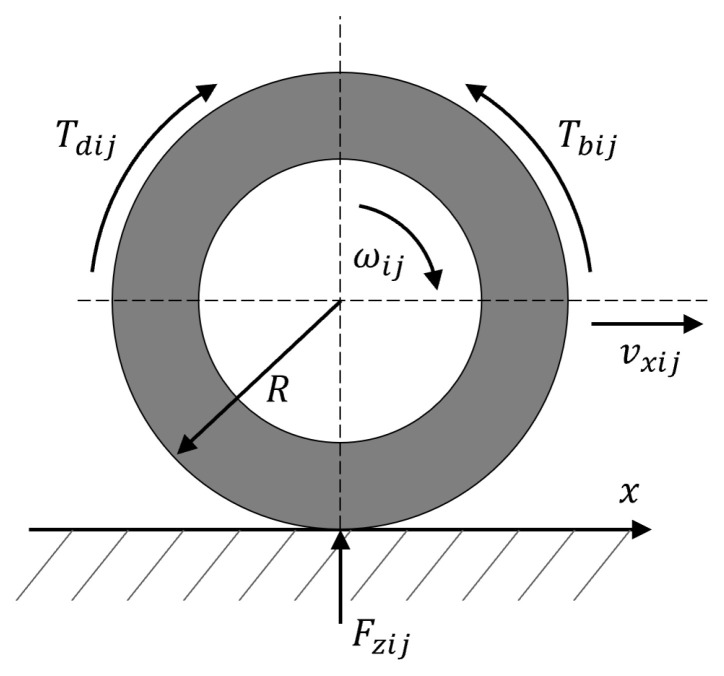
Rotational motion of the wheel.

The rotational motion of each wheel, illustrated in Figure 2, can be defined as follows [37]:

(4) $$I{\dot{\omega }}_{ij}=T_{dij}-T_{bij}-F_{xij}R-T_{fij}$$

where *I* is the inertial moment of the wheel, $\omega_{ij}$ is the angular velocity of each wheel, $T_{dij}$ is the driving torque, $T_{bij}$ is the braking torque, $T_{f}$ is the rolling resistance torque, $F_{zij}$ is the vertical load of the tire, and *R* is the wheel radius.

Rolling resistance arises from the friction occurring at the interface of the tire and the road as the wheel makes contact with the road surface. The rolling resistance torque can be formulated as:

(5) $$T_{fij}=fF_{zij}R$$

where *f* is the rolling resistance constant of the tire, and $F_{zij}$ is the vertical load of each tire.

The vertical load exerted on the tire fluctuates depending on the vehicle’s motion state. This load is typically delivered through a vehicle’s axle, with the vertical load of each wheel calculated by

(6) $$\left\{\begin{array}{c} F_{zfl}=\frac{mgl_{r}}{2L}-\frac{ma_{x}h_{cg}}{2L}-\frac{ma_{y}l_{r}h_{cg}}{d_{f}L}\\ F_{zfr}=\frac{mgl_{r}}{2L}-\frac{ma_{x}h_{cg}}{2L}+\frac{ma_{y}l_{r}h_{cg}}{d_{f}L}\\ F_{zrl}=\frac{mgl_{f}}{2L}+\frac{ma_{x}h_{cg}}{2L}-\frac{ma_{y}l_{f}h_{cg}}{d_{r}L}\\ F_{zrr}=\frac{mgl_{f}}{2L}+\frac{ma_{x}h_{cg}}{2L}+\frac{ma_{y}l_{f}h_{cg}}{d_{r}L} \end{array}\right.$$

where *g* is the acceleration due to gravity, $L=l_{f}+l_{r}$ is a wheelbase, and $h_{cg}$ is the distance from the vehicle’s center to the ground surface.

The handling stability analysis of a vehicle is greatly influenced by the mechanical characteristics of the tires. Thus, selecting an appropriate tire model is crucial. The tire force can be represented as a function of the slip angle of each tire $\alpha_{ij}$, slip rate $\lambda_{ij}$, and road friction coefficient μ, as expressed in Equation (7) [8]. However, the longitudinal and lateral tire forces exhibit significant non-linearity under driving conditions due to the complex interactions between the road surface and the tire. Hence, accurately simulating tire behavior during real driving remains challenging.

(7) $$\left\{\begin{array}{c} F_{xij}=f\left(\alpha_{ij},\lambda_{ij},\mu \right)\\ F_{yij}=g\left(\alpha_{ij},\lambda_{ij},\mu \right) \end{array}\right.$$

### 2.2. Reference Model

**Figure 3:**
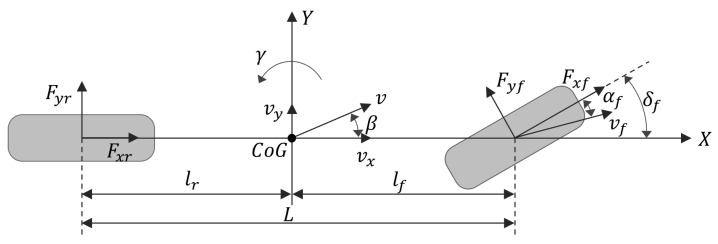
Linear 2-DOF vehicle model.

The linear 2-DOF vehicle dynamics model, shown in Figure 3, was selected to effectively explain the characteristics of the vehicle’s yaw motion based on driver intention. This model assumes only planar motion, with the roll angle, pitch angle, and vertical displacement all set to zero. Additionally, it does not account for the non-linearity and impact of steering or suspension. However, by assuming that this model operates within a linear range, it can better reflect the stable state of a vehicle and the driver’s intentions. Consequently, this linear 2-DOF dynamics model is utilized as a reference for obtaining the ideal yaw rate and lateral sideslip angle. Regarding the ideal yaw rate, used as a reference value for control, this study adopts the following expressions of the linear 2-DOF vehicle dynamics model [36]:

(8) $$\left\{\begin{array}{c} m\left(\dot{v_{y}}+v_{x}\gamma \right)=\left(k_{f}+k_{r}\right)\beta +\frac{1}{v_{x}}\left(l_{f}k_{f}-l_{r}k_{r}\right)\gamma -k_{f}\delta_{f}\\ I_{z}\dot{\gamma }=\left(l_{f}k_{f}-l_{r}k_{r}\right)\beta +\frac{1}{v_{x}}\left(l_{f}^{2}k_{f}-l_{r}^{2}k_{r}\right)\gamma -l_{f}k_{f}\delta_{f} \end{array}\right.$$

where $k_{f}$ and $k_{r}$ represent cornering stiffness coefficients of the front and rear wheels, respectively.

The lateral sideslip angle can be defined as the arctangent of the ratio of the longitudinal and lateral velocities; it varies according to the vehicle dynamics. Since this angle is generally very small, the tangent of the angle is approximately equal to the angle itself. Therefore, it can be approximated as [37]:

(9) $$\beta =\tan^{-1}\left(\frac{v_{y}}{v_{x}}\right)≅\frac{v_{y}}{v_{x}}$$

Using Equation (9), the linear 2-DOF vehicle dynamics model can be represented in terms of lateral and yaw movements with respect to the steering input as follows:

(10) $$\left\{\begin{array}{c} \dot{\beta }=\frac{k_{f}+k_{r}}{mv_{x}}\beta +\left(\frac{l_{f}k_{f}-l_{r}k_{r}}{mv_{x}^{2}}-1\right)\gamma -\frac{k_{f}}{mv_{x}}\delta_{f}\\ \dot{\gamma }=\frac{l_{f}k_{f}-l_{r}k_{r}}{I_{z}}\beta -\frac{l_{f}k_{f}}{I_{z}}\delta_{f}+\frac{{l_{f}}^{2}k_{f}+{l_{r}}^{2}k_{r}}{I_{z}v_{x}}\gamma \end{array}\right.$$

When a vehicle is driven in a steady state, its yaw rate and lateral sideslip angle should both be zero ($\dot{\beta }=0$ and $\dot{\gamma }=0$). Therefore, the ideal yaw rate and lateral sideslip angle in Equation (10) can be expressed as:

(11) $$\left\{\begin{array}{c} \beta_{d}=\left(\frac{l_{r}}{L}+\frac{ml_{f}v_{x}}{L^{2}k_{2}}\right)\frac{\delta_{f}}{1+Kv_{x}^{2}}\\ \gamma_{d}=\frac{v_{x}}{L\left(1+Kv_{x}^{2}\right)}\delta_{f} \end{array}\right.$$

Here, $K=\frac{m}{L^{2}}\left(\frac{l_{f}}{k_{f}}-\frac{l_{r}}{k_{r}}\right)$ represents the stability coefficient for the steady-state response of a vehicle.

The yaw rate may not always meet the tire adhesion limits due to factors such as high-speed driving, road conditions, and driving circumstances. An excessively high yaw rate may exceed the road adhesion limit, potentially leading to insufficient tire force. Therefore, tracking the desired yaw rate can be hazardous. In such cases, the required yaw rate must be limited by the tire–road friction coefficient.

Lateral acceleration, defined in terms of lateral dynamics and the lateral sideslip angle, is given by the following equation:

(12) $$a_{y}=\gamma v_{x}+\dot{v_{y}}=\gamma v_{x}+{\dot{v}}_{x}\tan\beta +\frac{v_{x}\dot{\beta }}{\sqrt{1+\tan^{2}\beta }\;}$$

The second and third terms in Equation (12) take a small value and account for approximately 15% of the total lateral acceleration [26]. Thus, the lateral acceleration is redefined as follows:

(13) $$a_{y}=\frac{\gamma v_{x}}{0.85}$$

Meanwhile, lateral acceleration is limited by the tire–road friction coefficient, as follows:

(14) $$a_{y}\leq \mu g$$

The yaw rate required by Equations (12)–(14) must satisfy the conditions specified as:

(15) $$\left|\gamma_{d}\right|\leq 0.85\left|\frac{\mu g}{v_{x}}\right|$$

Finally, the reference yaw rate can be re-expressed as:

(16) $$\left\{\begin{array}{c} \gamma_{d}=\min\left\{\left|\gamma_{d}\right|,\;\left|\gamma_{max}\right|\right\}sgn\left(\gamma_{d}\right)\\ \gamma_{max}=\frac{0.85}{v_{x}}\mu g \end{array}\right.$$

## 3. Controller Design

### 3.1. System Overview

A vehicle might encounter lateral instability if the driving motor fails to deliver adequate torque to the tires in extreme driving situations, such as sudden turns or abrupt maneuvers to avoid obstacles. Such circumstances can result in serious vehicle accidents. Therefore, this paper presents a novel method to enhance the yaw stability of vehicles, aiming to address this concern.

AFS and DYC are frequently employed as strategies for controlling the yaw stability of the vehicle. AFS generates an extra yaw moment by utilizing the lateral force of the tire. However, controlling the AFS system becomes infeasible when the lateral force of the tires reaches its maximum capacity. Consequently, this study introduced DYC, which utilizes an additional yaw moment for controlling the stability of the vehicle.

**Figure 4:**
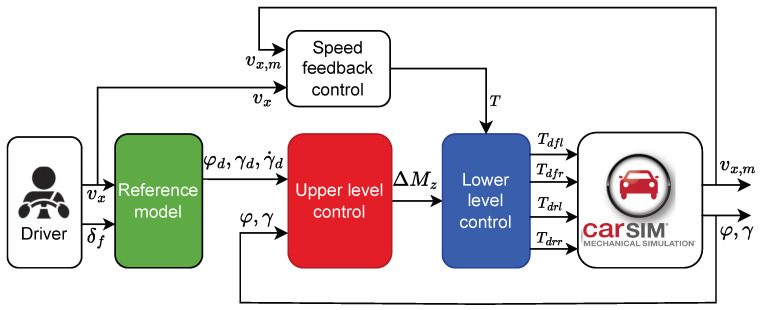
Overview of the DYC system.

The implemented DYC system utilizes a tiered structure as shown in Figure 4. An upper-level controller is developed by the NN-based NFTSMC control algorithm for obtaining the optimal extra yaw moment. Moreover, a lower-level controller employs an optimal distribution algorithm to ensure outstanding real-time control performance and effective torque distribution among the four wheels. Simultaneously, a speed feedback controller computes the necessary driving torque for the vehicle, enhancing the driving stability by ensuring the vehicle’s yaw stability through interactions between the controller layers.

### 3.2. Upper-Level Controller

#### 3.2.1. Non-Singular Fast Terminal SMC

Designing a sliding surface is a critical aspect of developing a sliding mode controller. In this study, an NFTSM surface has been chosen to achieve fast convergence, high tracking accuracy, and eliminate the singularity problem. Initially, the tracking errors of the yaw angle and yaw rate are defined as $e=\varphi -\;\varphi_{d}$ and $\dot{e}=\gamma -\gamma_{d}$, where $\varphi_{d}$ and $\gamma_{d}$ represent the desired values of φ and γ, respectively. Then, the NFTSM surface is designed as follows [26]:

(17) $$s=e+\lambda_{1}{\left|e\right|}^{p}sgn\left(e\right)+\lambda_{2}{\left|\dot{e}\right|}^{q}sgn\left(\dot{e}\right)$$

where $\lambda_{1}$ and $\lambda_{2}$ are positive constants, and *p* and *q* must fulfill the conditions 1<q<2, p>q. These values can be selected by the user to adjust the shape of the NFTSM surface.

The derivative of the NFTSM surface in Equation (17) can be calculated as follows:

(18) $$\dot{s}=\dot{e}+p\lambda_{1}{\left|e\right|}^{p-1}\dot{e}+q\lambda_{2}{\left|\dot{e}\right|}^{q-1}\ddot{e}=\dot{e}+p\lambda_{1}{\left|e\right|}^{p-1}\dot{e}+q\lambda_{2}{\left|\dot{e}\right|}^{q-1}\left(\dot{\gamma }-\dot{\gamma_{d}}\right)$$

Based on Equation (3), the yaw movement can be rewritten as:

(19) $$\begin{array}{cc} \dot{\gamma } & =\frac{1}{I_{z}}\left[l_{f}\left(F_{yfl}+F_{yfr}\right)\cos\delta_{f}+\frac{d_{f}}{2}\left(F_{yfl}-F_{yfr}\right)\sin\delta_{f}-l_{r}\left(F_{yrl}+F_{yrr}\right)+\Delta M_{z}\right]\\ \; & =\frac{1}{I_{z}}\left(F+\Delta M_{z}+D\right) \end{array}$$

where *F* represents the effect of the vehicle’s tire force, and $\Delta M_{z}$ represents the external yaw moment. They are defined as

(20) $$\left\{\begin{array}{c} F=l_{f}\left(F_{yfl}+F_{yfr}\right)\cos\delta_{f}+\frac{d_{f}}{2}\left(F_{yfl}-F_{yfr}\right)\sin\delta_{f}\;-l_{r}\left(F_{yrl}+F_{yrr}\right)\\ \Delta M_{z}=l_{f}\left(F_{xfl}+F_{xfr}\right)\sin\delta_{f}+\frac{df}{2}\left(F_{xrl}-F_{xrr}\right)+\frac{d_{f}}{2}\left(F_{xfl}-F_{xfr}\right)\cos\delta_{f} \end{array}\right.$$

Using Equation (19), Equation (18) can be restated as follows:

(21) $$\dot{s}=\dot{e}+p\lambda_{1}{\left|e\right|}^{p-1}\dot{e}+q\lambda_{2}{\left|\dot{e}\right|}^{q-1}\left(\frac{1}{I_{z}}\left(F+\Delta M_{z}+D\right)-\dot{\gamma_{d}}\right)$$

The additional yaw moment is formulated using NFTSM control as follows:

(22) $$\Delta M_{z}=\Delta M_{eq}+\Delta M_{re}$$

where $\Delta M_{eq}$ denotes the equivalent control law, and $\Delta M_{re}$ represents the reaching control law.

To reach the desired control performance, control inputs must be designed following the equivalent control law. To achieve this goal, the dynamics of the non-continuous sliding state must become zero. In other words, $\dot{s}=0$ must be fulfilled. To satisfy such a condition, the equivalent control law is designed as

(23) $$\Delta M_{eq}=I_{z}\left[\dot{\gamma_{d}}-\frac{{\left|\dot{e}\right|}^{2-q}}{q\lambda_{2}}\left(1+p\lambda_{1}{\left|e\right|}^{p-1}\right)sgn\left(\dot{e}\right)\right]-F$$

The equivalent control law may encounter control performance limitations owing to various external factors or parametric uncertainties. To minimize or eliminate these effects, the controller is designed using the reaching control law, which is a technique that assists the system state in approaching the sliding surface. Accordingly, a fast-reaching control law is designed to achieve fast convergence as follows [38]:

(24) $$\Delta M_{re}=I_{z}\left(-\eta_{1}sgn\left(s\right)-{\;\eta }_{2}{\left|s\right|}^{n_{2}}sgn\left(s\right)-{\;\eta }_{3}{\left|s\right|}^{n_{3}}sgn\left(s\right)\right)$$

where $\eta_{1}>0$, $\eta_{2}>0$, $\eta_{3}>0$, $n_{2}>1$, and $0<n_{3}<1$.

From Equations (23) and (24), the additional yaw moment is determined as follows:

(25) $$\begin{array}{cc} \Delta M_{z} & =\Delta M_{eq}+\Delta M_{re}\\ \; & =I_{z}\left[\dot{\gamma_{d}}-\frac{{\left|\dot{e}\right|}^{2-q}}{q\lambda_{2}}\left(1+p\lambda_{1}{\left|e\right|}^{p-1}\right)sgn\left(\dot{e}\right)\right]-F\\ \; & \;\;\;\;-I_{z}\left(\eta_{1}sgn\left(s\right)+\eta_{2}{\left|s\right|}^{n_{2}}sgn\left(s\right)+{\;\eta }_{3}{\left|s\right|}^{n_{3}}sgn\left(s\right)\right) \end{array}$$

From the control law in Equation (25), we observe that the fast-reaching control law is designed to counteract the uncertainty *D* and ensure rapid convergence of the sliding surface to the origin. However, this approach presents challenges in determining the correct upper boundary of *D* to establish the $\eta_{1}$ value for the control law, and it leads to a significant chattering phenomenon in the control signal. Additionally, the control law in Equation (25) requires consideration of the vehicle’s tire force *F*. The longitudinal and lateral tire forces are crucial for controlling and understanding the vehicle’s driving characteristics. However, accurately modeling tire behavior is challenging due to the highly complex and nonlinear interactions between the road surface and the tire, which vary with the driving state. Furthermore, tire models inherently contain uncertainties, and disturbances can arise from road and environmental conditions.

To address these issues comprehensively, we employ an RBFNN to approximate all components of *F* and *D*. With this design, the proposed control law does not require an explicit model of *F* and also does not mandate knowledge of the upper boundary value of the uncertainty *D*. The next subsections provide a detailed explanation of the proposed approach.

#### 3.2.2. Design of the NFTSMC Based on RBFNN

Conventional backpropagation neural network algorithms often exhibit slow learning rates and tend to converge to the local minima. In contrast, RBFNNs, which have recently been introduced for prediction purposes, feature dynamically adjustable weights, achieving exceptional approximation performance and global optimization capabilities. Additionally, they demonstrate excellent mapping capabilities, making them effective in function approximation, dynamic modeling, and system control. Moreover, their simple structure and fast convergence make them preferred for real-time applications.

The concept behind RBFNNs involves constructing a hidden layer using a radial basis function, such as Gaussian or logarithmic functions, as the activation function. Consequently, the input layer has a non-linear correlation with the hidden layer, whereas the connection between the hidden layer and the output layer is modeled linearly [31]. Utilizing these attributes, RBFNNs can transform input data into higher-dimensional data. Furthermore, RBFNNs can mitigate the chattering phenomenon through gain tuning of the control reaching law in a sliding mode controller [31].

**Figure 5:**
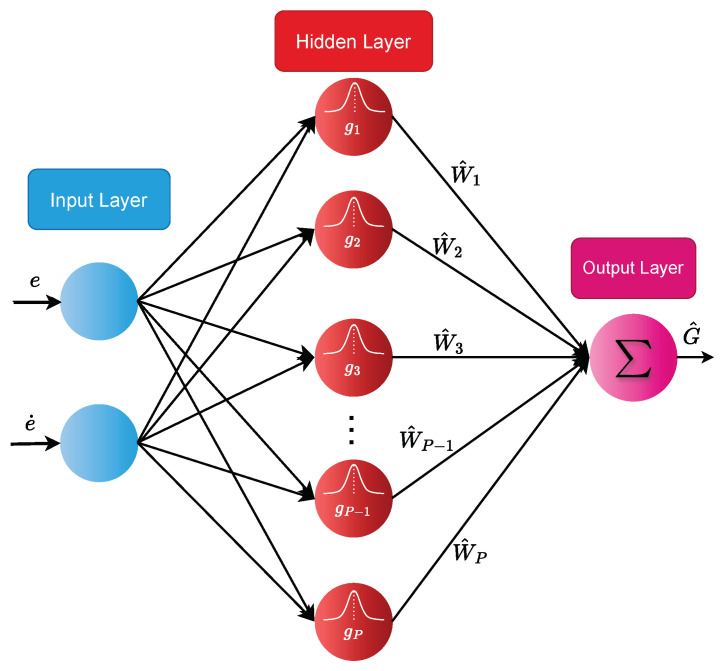
The structure of RBFNN.

This study incorporates an RBFNN to estimate all effects of the vehicle’s tire force and uncertainty, expressed as G=F+D. As shown in Figure 5, a 2-P-1 RBFNN configuration is used, comprising an input layer with 2 inputs (*e* and $\dot{e}$), a hidden layer with *P* neuron nodes using the Gaussian function as the activation function, and an output.

An approximation of the function *G* can be achieved precisely through the utilization of an RBFNN as follows:

(26) $$G=F+D=W^{T}g\left(x\right)+\varepsilon$$

where $x={\left[e\;\;\;\dot{e}\right]}^{T}$ represents the network input that comprises the yaw rate error and its derivative, *W* is the weight, ε represents the approximation error of the RBFNN bounded by $\left|\varepsilon \right|\leq {\bar{\eta }}_{1}$, and $g\left(x\right)={\left[g_{1},g_{2},...,g_{P}\right]}^{T}$ is the output of the Gaussian function, defined as

(27) $$g_{i}=\left(\exp\frac{{∥x-c_{ij}∥}^{2}}{b_{i}^{2}}\right)$$

Here, $i=\left\{1,2\right\}$ is related to the input vector, and $j=\left\{1,2,\cdots ,\;P\right\}$ represents the number of hidden layer nodes, c denotes the center point of the Gaussian function, and b represents the width vector of the Gaussian function, as specified in the following:

(28) $$\left\{\begin{array}{c} c=\left[c_{ij}\right]=\left[\begin{array}{ccc} c_{11} & c_{12} & \begin{array}{cc} \cdots & c_{1P} \end{array}\\ c_{21} & c_{22} & \begin{array}{cc} \cdots & c_{2P} \end{array} \end{array}\right]\\ b=\;\left[b_{j}\right]={\left[\begin{array}{ccc} b_{1}, & \cdots , & b_{P} \end{array}\right]}^{T} \end{array}\right.$$

Then, the RBFNN’s output, $\hat{G}$, is computed as

(29) $$\hat{G}\left(x\right)={\hat{W}}^{T}g\left(x\right)$$

in which $\hat{W}$ denotes the estimated weight vector of *W*.

By using the estimated value $\hat{G}$ from the RBFNN, the equivalent control law in Equation (23) is redesigned as follows:

(30) $$\begin{array}{cc} \Delta M_{eq,\;new} & =I_{z}\left[\dot{\gamma_{d}}-\frac{{\left|\dot{e}\right|}^{2-q}}{q\lambda_{2}}\left(1+p\lambda_{1}{\left|e\right|}^{p-1}\right)sgn\left(\dot{e}\right)\right]-\hat{G} \end{array}$$

Ultimately, the additional yaw moment calculated based on the proposed NN-based NFTSMC is designed as follows:

(31) $$\begin{array}{cc} \Delta M_{z} & =\Delta M_{eq,\;new}+\Delta M_{re,\;new}\\ \; & =I_{z}\left[\dot{\gamma_{d}}-\frac{{\left|\dot{e}\right|}^{2-q}}{q\lambda_{2}}\left(1+p\lambda_{1}{\left|e\right|}^{p-1}\right)sgn\left(\dot{e}\right)\right]-\hat{G}\\ \; & \;\;\;\;-I_{z}\left({\bar{\eta }}_{1}sgn\left(s\right)+\eta_{2}{\left|s\right|}^{n_{2}}sgn\left(s\right)+{\;\eta }_{3}{\left|s\right|}^{n_{3}}sgn\left(s\right)\right)\\ \; & =I_{z}\left[\dot{\gamma_{d}}-\frac{{\left|\dot{e}\right|}^{2-q}}{q\lambda_{2}}\left(1+p\lambda_{1}{\left|e\right|}^{p-1}\right)sgn\left(\dot{e}\right)\right]-{\hat{W}}^{T}g\left(x\right)\\ \; & \;\;\;\;-I_{z}\left({\bar{\eta }}_{1}sgn\left(s\right)+\eta_{2}{\left|s\right|}^{n_{2}}sgn\left(s\right)+{\;\eta }_{3}{\left|s\right|}^{n_{3}}sgn\left(s\right)\right) \end{array}$$

where ${\bar{\eta }}_{1}$ is a small positive constant and the other parameters are defined as in Equation (24).

The weight update rule of the NN is designed as

(32) $$\dot{\hat{W}}=Q^{-1}g\left(x\right)\theta s$$

where $\theta =q\lambda_{2}{\left|\dot{e}\right|}^{q-1}>0\;\forall \dot{e}\neq 0$, and *Q* is a positive coefficient.

**Figure 6:**
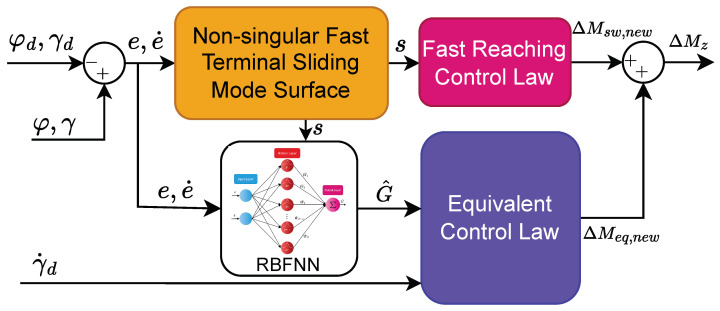
The schematic of upper-level control.

The schematic of the upper-level control system is shown in Figure 6. The upper-level controller receives input signals such as the desired values ($\varphi_{d}$, ${\dot{\gamma }}_{d}$, ${\ddot{\gamma }}_{d}$) and the actual values (φ, $\dot{\gamma }$). From these signals, tracking error values (*e*, $\dot{e}$) are calculated to serve the calculation of the NFTSM surface and provide input to the RBFNN. Based on the calculated sliding surface *s*, the fast-reaching control law can be determined, and the weight values of the NN can be updated. The output of the NN, denoted as $\hat{G}$, is then used to calculate the equivalent control law. Finally, the proposed additional yaw moment is formed by summing the values of the equivalent control law and the fast-reaching control law.

From the proposed control law in Equation (31), we can see that an RBFNN is used to approximate the entire vehicle tire force model and the uncertainty components. Therefore, the proposed control law does not require calculating the vehicle’s tire force model, which is difficult to calculate accurately in a real system. Furthermore, in the control law (31), only a small sliding gain value ${\bar{\eta }}_{1}$ is needed to compensate for the estimation error of the NN. This value of ${\bar{\eta }}_{1}$ can be much smaller than the value of $\eta_{1}$ in Equation (25). With this approach, the proposed control law can significantly reduce the chattering behavior in the control signal.

**Remark 1.**  *The control parameters of the proposed method must be selected according to the conditions specified in Equations (17), (24), (31), and (32) to ensure the stability of the control system. These parameters should be chosen as follows:* $\lambda_{1}$ *and* $\lambda_{2}$ *should be greater than 0;* $\eta_{2}$ *and* $\eta_{3}$ *should be greater than 0; q should be chosen between 1 and 2;* *p* *should be greater than* *q;* $n_{2}$ *should be greater than 1;* $n_{3}$ *should be between 0 and 1;* $\eta_{1}$ *should be a small positive number; and Q should be a positive number. Additionally, to achieve the expected performance, these control parameters can be fine-tuned through repeated testing and output verification.*

#### 3.2.3. Stability Analysis

To validate the stability of the proposed controller, the Lyapunov candidate is chosen as follows:

(33) $$V=\frac{1}{2}s^{2}+\frac{1}{2}Q{\tilde{W}}^{T}\tilde{W}$$

where $\tilde{W}=W-\hat{W}$ denotes the weight error of NN.

Differentiating Equation (33) with respect to time yields

(34) $$\dot{V}=s\dot{s}+Q{\tilde{W}}^{T}\dot{\tilde{W}}$$

Substituting $\dot{s}$ from Equation (21) into Equation (34), we obtain

(35) $$\dot{V}=s\left(\dot{e}+p\lambda_{1}{\left|e\right|}^{p-1}\dot{e}+q\lambda_{2}{\left|\dot{e}\right|}^{q-1}\left(\frac{1}{I_{z}}\left(G+\Delta M_{z}\right)-\dot{\gamma_{d}}\right)\right)-Q{\tilde{W}}^{T}\dot{\hat{W}}$$

Applying the proposed control law in Equation (31) and the NN weight update law in Equation (32) to Equation (35), we obtain

(36) $$\begin{array}{cc} \dot{V} & =s\left[\theta \left({\tilde{W}}^{T}g\left(x\right)+\varepsilon -{\bar{\eta }}_{1}sgn\left(s\right)-\eta_{2}{\left|s\right|}^{n_{2}}sgn\left(s\right)-\eta_{3}{\left|s\right|}^{n_{3}}sgn\left(s\right)\right)\right]-{\tilde{W}}^{T}Q\dot{\hat{W}}\\ \; & =s\left[\theta \left(\varepsilon -{\bar{\eta }}_{1}sgn\left(s\right)-\eta_{2}{\left|s\right|}^{n_{2}}sgn\left(s\right)-\eta_{3}{\left|s\right|}^{n_{3}}sgn\left(s\right)\right)\right]+{\tilde{W}}^{T}\left(g\left(x\right)\theta s-Q\dot{\hat{W}}\right)\\ \; & \leq \theta \left(\left(|\varepsilon \right|-{\bar{\eta }}_{1})|s|-\eta_{2}{\left|s\right|}^{n_{2}+1}-\eta_{3}{\left|s\right|}^{n_{3}+1}\right)\\ \; & \leq -\theta \left(\eta_{2}{\left|s\right|}^{n_{2}+1}+\eta_{3}{\left|s\right|}^{n_{3}+1}\right)\leq 0 \end{array}$$

From Equations (33) and (36), we can see that V>0 and $\dot{V}<0$. Therefore, we can conclude that the control system is stable.

### 3.3. Lower-Level Controller

In generating an additional yaw moment calculated using the proposed NN-based NFTSM controller, distributing torque reasonably among the four wheels is crucial. Methods such as average [39], optimal [40], and dynamic vertical load distributions [41] are frequently employed for this purpose. When a vehicle is turning, there is a load distribution of longitudinal and lateral forces. This is also closely related to the friction coefficient of the road surface. This study thus adopted a strategy to allocate the torque based on the vertical dynamic load ratio of the wheels. With an increase or decrease in the vertical dynamic load, the required torque increases or decreases correspondingly. Given that the longitudinal force obtained from the proposed upper-level controller can be distributed according to the vertical load ratio of each wheel, the longitudinal force of each wheel can be efficiently utilized.

First, the relation between the longitudinal force and yaw moment of each tire is specified as

(37) $$\left\{\begin{array}{c} F_{xfl}=\frac{F_{zfl}}{F_{z}}\cdot \frac{\Delta M_{z}}{a\sin\delta_{f}-d_{f}/2\cos\delta_{f}}\\ F_{xfr}=\frac{F_{zfr}}{F_{z}}\cdot \frac{\Delta M_{z}}{a\sin\delta_{f}+d_{f}/2\cos\delta_{f}}\\ F_{xrl}=-\frac{F_{zrl}}{F_{z}}\cdot \frac{\Delta M_{z}}{2d_{r}}\\ F_{xrr}=\frac{F_{zrr}}{F_{z}}\cdot \frac{\Delta M_{z}}{2d_{r}} \end{array}\right.$$

Based on Equation (37), the driving torques of four wheels calculated from the correlation with the longitudinal force are expressed as follows:

(38) $$\left\{\begin{array}{c} T_{dfl}=\frac{F_{xfl}}{R}\\ T_{dfr}=\frac{F_{xfr}}{R}\\ T_{drl}=\frac{F_{xrl}}{R}\\ T_{drr}=\frac{F_{xrr}}{R} \end{array}\right.$$

## 4. Simulation Results

To validate the effectiveness of the proposed DYC system, simulation experiments under different operating conditions were performed in this study. Additionally, to highlight the robustness of the proposed method, its control performance is compared with the SMC, NFTSMC, and without control.

The additional yaw moment control law of SMC is given by

(39) $$\Delta M_{z}=I_{z}\left[\dot{\gamma_{d}}-c\dot{e}-\eta_{1}sgn\left(s\right)-\eta_{2}s\right]-F$$

where $s=\dot{e}+ce$ represents the linear sliding mode surface, c>0, $\eta_{1}>0$, and $\eta_{2}>0$.

The additional yaw moment control law of NFTSMC is designed by

(40) $$\Delta M_{z}=I_{z}\left[\dot{\gamma_{d}}-\frac{{\left|\dot{e}\right|}^{2-q}}{q\lambda_{2}}\left(1+p\lambda_{1}{\left|e\right|}^{p-1}\right)sgn\left(\dot{e}\right)-\eta_{1}sgn\left(s\right)-\eta_{2}s\right]-F$$

where *s* is defined as Equation (17), $\eta_{1}>0$, and $\eta_{2}>0$.

The simulation experiments were carried out through co-simulation involving CarSim and MATLAB/Simulink, which were used to configure the simulation environments encompassing diverse driving conditions. In this study, a 4WID B-class hatchback model from CarSim software was employed as a test vehicle, with detailed vehicle parameters provided in Table 1 [32].

To evaluate the efficacy of the suggested lateral stability control method, extreme simulation scenarios were formulated using two steering angle inputs (step input and sinusoidal input). The vehicle underwent testing on a level surface of 1 $km^{2}$ where the influence of air resistance and slope resistance was disregarded, and only road friction resistance was taken into account. The simulation conditions were set with a desired vehicle speed $v_{x}$ of 80 km/h, road friction coefficient μ of 0.85, and rolling resistance coefficient of 1.

To facilitate the evaluation of the performance of the control methods, the root mean square error (RMSE) and the error peak value for the yaw rate were calculated and presented in Table 2 and Table 3.

### 4.1. Simulation Results of Step Steering Angle Input

**Figure 7:**
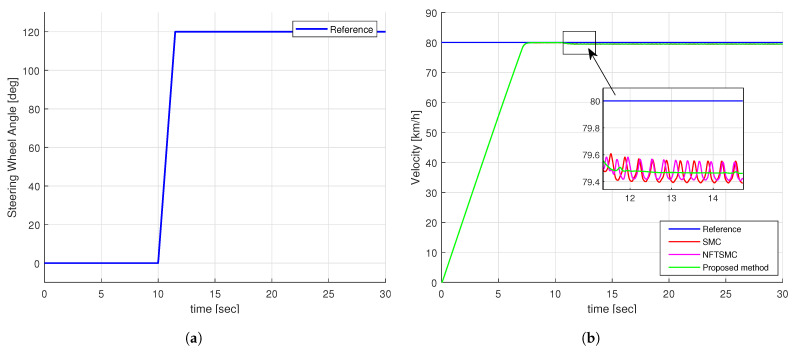
The simulation input of step test: (**a**) Steering wheel angle, (**b**) Velocity of vehicle.

Figure 7a illustrates the step-shaped input for the steering wheel angle. Notably, at the 10-second mark, the angle surges rapidly from 0 to 120 degrees, expressing an extreme scenario. Concurrently, Figure 7b presents the specified speed and the actual speed of the vehicle under various control methods. It is evident from Figure 7b that despite some discrepancies, all control methods demonstrate the capability to maintain speed tracking effectively.

**Figure 8:**
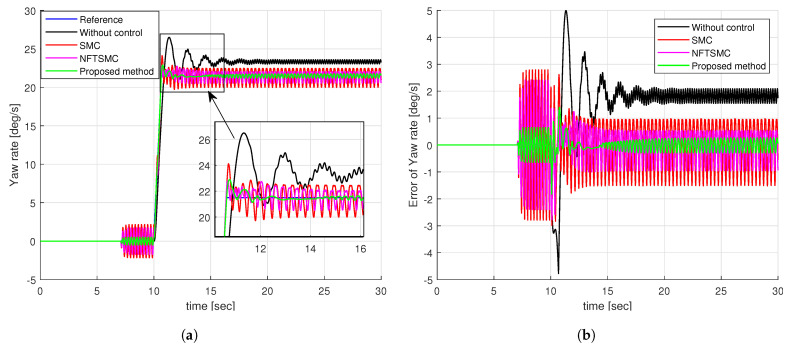
Simulation results of step input response: (**a**) The tracking performance of yaw rate, (**b**) the tracking error of yaw rate.

Figure 8a illustrates the yaw rate tracking performance of different control methods. We can see that it is very difficult for the vehicle to track the desired yaw rate without control. The SMC, NFTSMC, and proposed methods can all track the desired yaw rate as shown in Figure 8a. By observing the enlarged figure in Figure 8a, we can easily see that the proposed method provides better tracking of the desired yaw rate than the SMC and NFTSMC methods. Notably, the yaw rates of the SMC and NFTSMC methods are significantly shaken compared to the proposed method.

**Figure 9:**
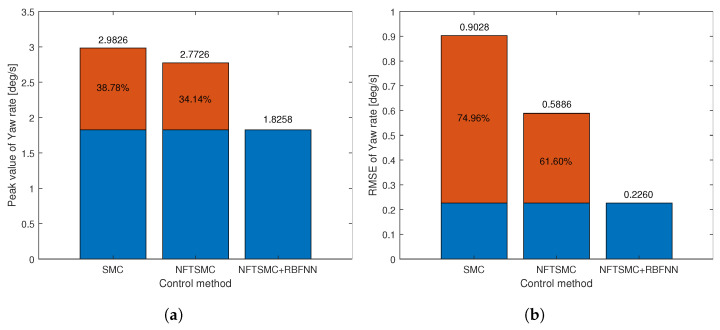
The objective performance indicator of step input response: (**a**) Peak value, (**b**) RMSE.

For a more detailed analysis of the tracking performance of control methods, we can observe the tracking error of the yaw rate presented in Figure 8b and the results of calculating the peak value and RMSE of the tracking error of the yaw rate in Table 2. We can easily see that the tracking accuracy of NFTSMC is slightly better than that of the SMC method, while the accuracy of the proposed method is significantly better than all remaining methods. Figure 9 shows the tracking accuracy improvement and peak value reduction of the proposed strategy compared to the SMC and NFTSMC strategies. Observing Figure 9, we see that the maximum yaw rate error of the proposed algorithm is 1.8258 deg/s. This value is 38.78% and 31.14% lower than 2.9826 deg/s and 2.7726 deg/s, respectively, which are the maximum yaw rate errors of the SMC and NFTSMC control methods. Additionally, the RMSE of the proposed method decreases by 74.96% and 61.60% compared to that of SMC and NFTSMC, respectively.

**Figure 10:**
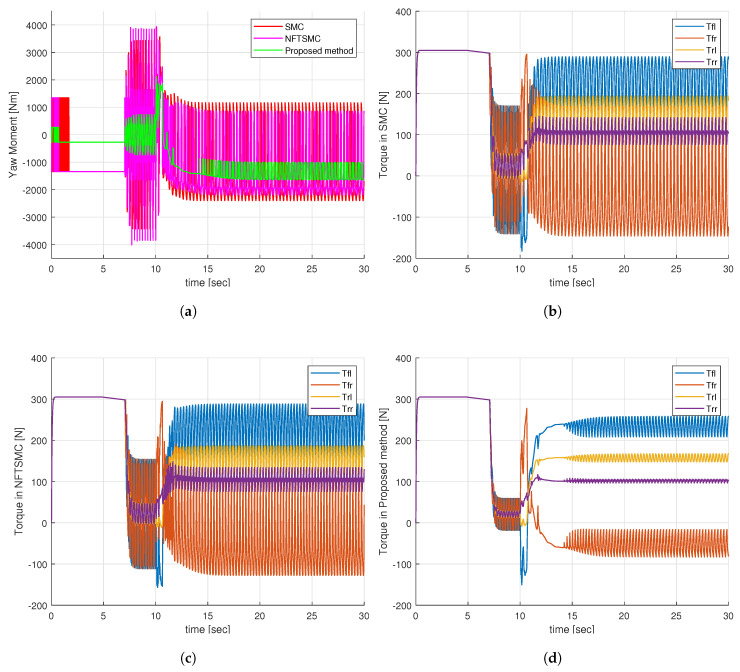
Simulation results of step input response: (**a**) Additional yaw moment, (**b**) Control torques—SMC, (**c**) Control torques—NFTSMC, (**d**) Control torques—proposed method.

Observing Figure 10a, it is evident that the additional yaw moment generated by the SMC and NFTSMC methods exhibits severe chattering behavior, whereas the proposed method greatly reduces this chattering effect. This improvement is achieved because the proposed method requires only a small sliding gain value, ${\bar{\eta }}_{1}$, to compensate for the approximation error of the NN. In contrast, the SMC and NFTSMC methods must use a large sliding gain value, which needs to be greater than the upper boundary of the uncertainty components. Upon analyzing the additional yaw torques in Figure 10a, it becomes apparent that the control torques at the four wheels for the SMC and NFTSMC methods exhibit substantial chattering, as indicated in Figure 10b,c. Notably, the control torques at the four wheels for the proposed method demonstrate a significant reduction in chattering behavior, as depicted in Figure 10d.

### 4.2. Simulation Results of Sine Steering Angle Input

**Figure 11:**
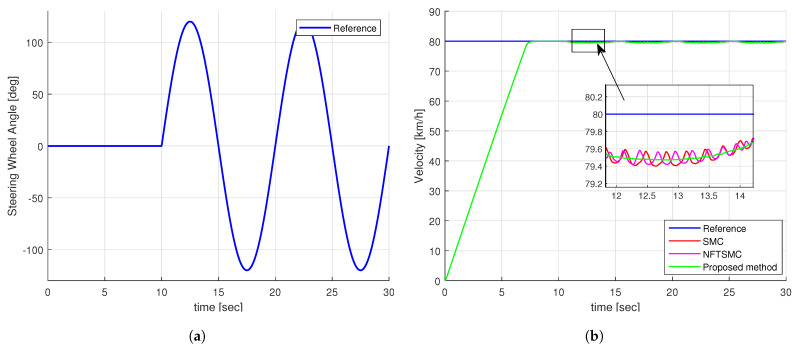
The simulation input of sine test: (**a**) Steering wheel angle, (**b**) Velocity of vehicle.

The sine-wave steering angle is often used as an input to test vehicle lateral stability in extreme conditions. As depicted in Figure 11a, a sinusoidal steering wheel angle with a magnitude of 120 degrees was set for this experiment. Figure 11b depicts the vehicle speed under different control methods. Looking at Figure 11b, we can see that all control methods can maintain the ability to track the required speed of the vehicle.

**Figure 12:**
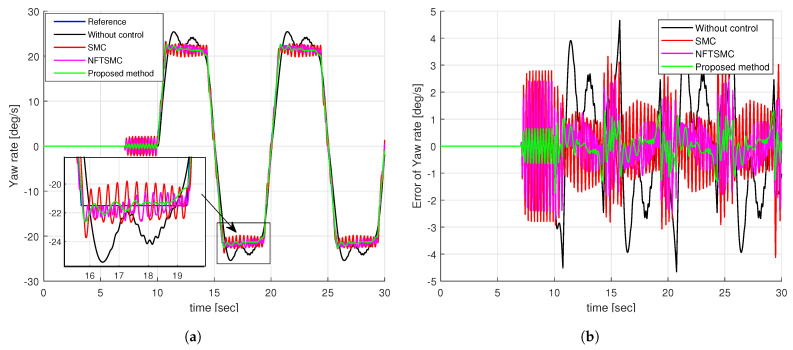
The simulation results of the sine input response: (**a**) The tracking performance of yaw rate, (**b**) the tracking error of yaw rate.

**Figure 13:**
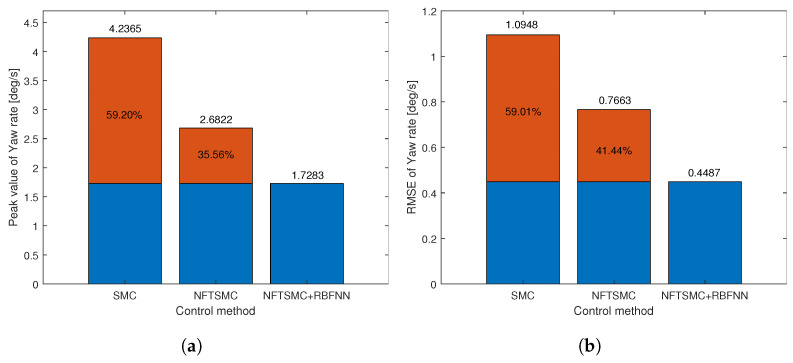
The objective performance indicator of sine input response: (**a**) Peak value, (**b**) RMSE.

Figure 12a presents the yaw rate tracking performance of the control methods for sinusoidal steering angle input, while Figure 12b illustrates the yaw rate tracking errors of the control methods. It is evident from Figure 12a that it is very difficult for the vehicle to track the desired yaw rate without control. However, the remaining control methods demonstrate the capability to track the desired yaw rate effectively. By observing the tracking error in Figure 12a, we can easily see that the proposed control method provides the best yaw rate tracking accuracy compared to the other methods. For a more detailed analysis, the calculated results of RMSE and peak error value are presented in Table 3, and their illustration is shown in Figure 13. The RMSE of the proposed method is 0.4487 deg/s, significantly improving accuracy by 59.01% compared to SMC and 41.44% compared to NFTSMC, as depicted in Figure 13a. Additionally, the peak error value of the proposed method is 1.7283 deg/s, which is the smallest compared to the other methods. Meanwhile, the peak error value of the NFTSMC method is 2.6822 deg/s, which is smaller than the error value of the SMC method 4.2365 deg/s. Looking at Figure 13b, it is evident that the peak error value of the proposed method is reduced by 59.20% compared to the SMC method and 35.56% compared to the NFTSMC method.

**Figure 14:**
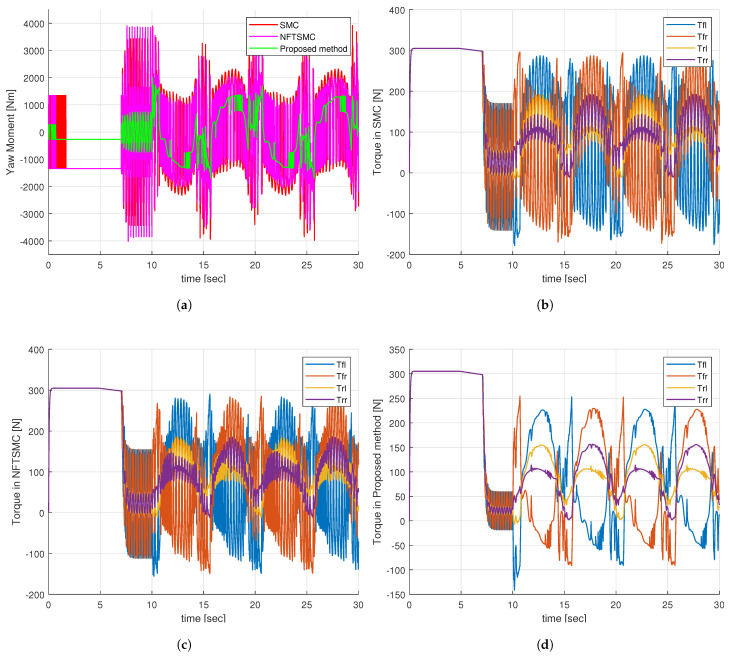
Simulation results of sine input response: (**a**) Additional yaw moment, (**b**) Control torques—SMC, (**c**) Control torques—NFTSMC, (**d**) Control torques—proposed method.

The comparison of the additional yaw torque of the control methods is depicted in Figure 14a, and the control torques at the four wheels of the control methods are presented in Figure 14b–d. Similar to the case of step steering angle input, the additional yaw moment generated by the proposed method has significantly reduced chattering behavior compared to the SMC and NFTSMC methods, as shown in Figure 14a. Furthermore, the control torques at the four wheels of the vehicle generated by the proposed method shown in Figure 14d are also smoother than the SMC and NFTSMC methods, as shown in Figure 14b,c. The smoother control torques from the proposed method contribute to diminishing the adverse impact on the motor, thereby extending the lifespan of the vehicle’s mechanical systems.

Through analyzing the results above, it becomes evident that the proposed control method provides superior yaw rate tracking performance compared to the remaining methods. Specifically, it achieves the highest yaw rate tracking accuracy and exhibits the smallest peak tracking error values when compared to the SMC and NFTSMC strategies. Additionally, the control torque signals at the four wheels of the proposed method significantly reduce the chattering phenomenon compared to the other methods. This reduction in chattering not only enhances vehicle stability but also contributes to extending the lifespan of the vehicle’s mechanical systems, providing substantial benefits in terms of durability and performance.

## 5. Conclusions

This study introduces a new DYC architecture strategy for improving the yaw stability of 4WID electric vehicles. The proposed DYC system adopts a hierarchical control approach, where an NN-based NFTSMC method in the upper-level controller generates an optimal additional yaw moment, while the lower-level controller distributes optimal torque based on the vertical load ratio. To assess the control effectiveness of the proposed DYC system, its performance has been compared to DYC systems based on conventional SMC and NFTSMC methods across various extreme driving conditions. The simulation results underscore the superiority of the proposed method, showcasing improved vehicle yaw rate tracking accuracy and a substantial reduction in control torque chattering. In the future, we plan to validate the proposed controller through on-road testing, thereby bridging the gap between simulation and real-world performance. Additionally, we aim to explore the development of DYC systems grounded in real-time optimization techniques. These efforts will not only validate the practical applicability of the proposed DYC system under real-world road conditions but also pave the way for the advancement of more robust and effective control mechanisms.

## Figures and Tables

**Table 1:** Vehicle configuration.

Parameter	Unit	Symbol	Value
Mass of Vehicle	kg	*m*	1134
Wheelbase	mm	*L*	2600
Distance from CoG to front axle	mm	$l_{f}$	1040
Distance from CoG to rear axle	mm	$l_{r}$	1560
Front wheel tread	mm	$d_{f}$	1040
Rear wheel tread	mm	$d_{r}$	1560
Wheel radius	mm	*R*	1485
Yaw rotational moment of inertia	$kg\cdot m^{2}$	$I_{z}$	1343.1

**Table 2:** The simulation results of the step input response.

Method	Peak Value [deg/s]	RMSE [deg/s]
SMC	2.9826	0.9028
NFTSMC	2.7726	0.5886
Proposed method	1.8258	0.2260

**Table 3:** The simulation results of the sine input response.

Method	RMSE [deg/s]	Peak Value [deg/s]
SMC	1.0948	4.2365
NFTSMC	0.7663	2.6822
Proposed method	0.4487	1.7283

## Data Availability

The data are contained within the article.

## Abbreviations

The following abbreviations are used in this manuscript:

4WID	Four-wheel independent drive
ABS	Anti-lock braking system
AFS	Active front steering
DOF	Degree-of-freedom
DYC	Direct yaw-moment control
EV	Electric vehicle
PID	Proportional–integral–derivative
LQR	Linear quadratic regulator
MPC	Model predictive control
SMC	Sliding mode control
TSMC	Terminal SMC
NTSMC	Non-singular TSMC
NFTSMC	Non-singular fast TSMC
NN	Neural network
RBFNN	Radial basis function NN
RMSE	Root mean square error
